# Drug levels of VEDOLIZUMAB in patients with pediatric-onset inflammatory bowel disease in a real-life setting

**DOI:** 10.1007/s00431-023-05255-y

**Published:** 2023-10-25

**Authors:** Maria Hemming-Harlo, Laura Merras-Salmio, Anne Nikkonen, Kaija-Leena Kolho

**Affiliations:** Children’s Hospital, Helsinki University Hospital HUS and University of Helsinki, Stenbäckinkatu 11 FI-00290 , Helsinki, Finland

**Keywords:** Antibody, Children, Crohn´s disease, Integrin, Ulcerative colitis

## Abstract

Vedolizumab (VDZ) is used off-label in pediatric inflammatory bowel disease (PIBD). There are less data on drug levels to achieve and maintain remission in children. We aimed to study vedolizumab (VDZ) trough levels in a pediatric population in a real-life setting. We traced 50 patients with PIBD receiving VDZ treatment at our hospital, reviewed their treatment protocol, trough levels, and antidrug antibodies, and compared those to fecal calprotectin (FC) levels and achievement of corticosteroid-free maintenance therapy (CF). VDZ trough level was available from 198 samples during a median follow- up of 12.6 months. Proceeding to maintenance therapy was associated with a decline in FC but not with VDZ trough levels that were comparable between patients with FC < 100 μg/g (remission), 100–1000 μg/g, or > 1000 μg/g at 3 months (mean levels of 36.8, 28.6, and 27 μg/mL, respectively p = 0.188). At 3 months, patients achieving CF (41%) and those on corticosteroids had comparable VDZ trough levels (33 vs. 27.5 μg/mL, respectively). At 6 months, the trough level was similar in groups with FC < 100 μg/g or FC > 1000 μg/g (31.5 and 27.6 μg/mL, p = 0.859). Treatment intensification did not improve the achieved CF at 12 months. None developed drug antibodies nor discontinued the therapy for an adverse event.

*Conclusion*: VDZ was a well-tolerated and safe biologic treatment. A positive response on gut inflammation after induction predicted proceeding to maintenance therapy whereas trough levels did not. A VDZ trough level associated with clinical remission or continuing with VDZ treatment could not be determined.

**Table:** 

**What is Known:** *• In pediatric inflammatory bowel disease, vedolizumab is still in off-label use.* *• The results on the relationship between drug levels of vedolizumab and clinical remission in pediatric patients are contradictory.*	
**What is New:** *• This real-life setting in pediatric-onset inflammatory bowel disease showed no benefit of therapy enhancement during a median follow-up of one year.* *• Trough levels of vedolizumab were not associated with therapy outcomes.*

**Supplementary Information:**

The online version contains supplementary material available at 10.1007/s00431-023-05255-y.

## Introduction

As the number of patients with pediatric-onset inflammatory bowel diseases (PIBD), including Crohn disease (CD), ulcerative colitis (UC), and inflammatory bowel disease unclassified (IBD-U), has increased remarkably, more real-life data on therapies used off-label in children are needed especially for patients who do not respond or lose their response to anti-tumor necrosis factor (TNFα) [1–5].

VDZ is a monoclonal immunoglobulin G1 antibody directed against a4b7 integrin that is shown to reduce intestinal inflammation by multiple mechanisms and by restricting b7 + effector response to the intestinal epithelium [6–8]. VDZ was registered for adult patients with IBD in 2014, showing efficacy in both CD and UC [9–11]. In PIBD, VDZ is still in off-label use. The reported efficacy rates have varied [12–14]. So far, there is only a limited number of studies concerning the dosing and trough levels of VDZ for achieving and maintaining remission. The VDZ trough levels in adults are based on GEMINI trials and a low number of real-world cohorts [9, 10] and in clinical practice these levels are targeted in pediatric patients as well. Previous publications, however, have reported contradictory results on the relationship between VDZ trough levels and clinical remission [15–17].

In this study, we aimed to determine the role of VDZ trough levels in clinical outcomes defined by achieved corticosteroid-free therapy (CF) and a decrease of fecal calprotectin (FC) in a pediatric population in a real-life setting.

## Material and methods

### Patient population and study design

The data were collected retrospectively on all children with PIBD introduced to their first VDZ treatment protocol at our tertiary care hospital between January 2017 and June 2020. The patients were identified from a clinical register including all patients with PIBD treated with biologics. The study surveillance continued until the VDZ treatment ended, or the patient was transferred into adult healthcare, or by the end of the year 2020. All therapeutic decisions were made at the discretion of the pediatric gastroenterologists applying the clinical symptom index [18], FC, blood inflammatory markers and trough levels, and drug antibodies routinely measured at the time of infusion and patient history.

### Outcome measurements

The patient data were analyzed at baseline and week 6, months 3, 6, 12, and 24. We reviewed data on patient age, sex, the subtype of PIBD, disease duration, previous biologic, immunosuppressive, or other medication, and VDZ trough levels and FC values.

The standard protocol consisted of induction infusions of VDZ at weeks 0, 2, and 6, followed by maintenance therapy at every 8 weeks. VDZ was prescribed at the clinicians´ discretion, generally 5 mg/kg up to a maximum of 300 mg.

The primary outcome was the achieved corticosteroid-free remission (CF) defined as the absence of oral glucocorticoids use. Biological remission was defined as FC decrease to less than 100 μg/g [19, 20]. Also, a 50% decline in FC was considered significant.

### Laboratory analyses

VDZ trough levels were routinely determined using enzyme-linked immunosorbent assay (ELISA) performed by Sanquin, Netherlands. FC was quantitated routinely with an ELISA kit from Calpro AS (Calpro/Calprolab, Oslo, Norway) as described earlier [21].

### Statistical analysis

The data are presented as a median and interquartile range (IQR) unless otherwise stated. We used Fisher's exact test to determine differences in binary variables. The non-parametric Mann-Whitney test was used to compare continuous variables between the groups as appropriate. Kaplan-Meier curves were evaluated using a log-rank test. The software used for the analysis was Graph Pad Prism version 7.0 (GraphPad Software). P-values of < 0.05 were considered as significant.

### Ethical considerations

This was a register-based study. According to Finnish legislation, ethical approval, or informed consent was not needed as the patients were not contacted.

## Results

We traced 50 patients with PIBD treated with VDZ. Of those, 28% were diagnosed with CD, 34% with UC, and 38% with IBD-U. Of all patients, 74% (37/50 patients) were treated with at least one anti-TNFα agent before VDZ, 18% (9/50 patients) had been treated with at least 2 anti-TNFα agents, and 10% (5/50 patients) had received ustekinumab (Table 1). All outcomes were solely related to VDZ treatment as no other medication (biologic, methotrexate, or azathioprine) was started during the observation period with a median duration of follow-up of 12.6 months.

**Table 1:** Characteristics of the pediatric patients with inflammatory bowel disease

		All patients (N = 50)	Crohn disease (CD) (N = 14)	Ulcerative colitis (UC) (N = 17)	Unclassified IBD (IBD-U) (N = 19)
Age at diagnosis, years, median (range)		12.1	12.4	12.4	12.3
		(3.2–16.1)	(3.8–15.5)	(8.7–15.3)	(3.2–16.1)
Age at start of vedolizumab, median (range)		15.3	15.1	15.4	14.6
		(4.6–19.1)	(4.6–18.8)	(12.4–18.5)	(7.6–19.1)
Disease duration, years, median (range)		2.6	2.3	1.6	2.4
		(0.3–12.6)	(0.8–12.6)	(0.3–6.5)	(0.4–11.6)
Location CD, n (%)	L1 distal 1/3 ileum $\pm$ cecal disease		0		
	L2 colitis		3 (21)		
	L3 ileocolitis		6 (43)		
	L3 + L4a upper disease		5 (36)		
Behavior CD, n (%)	B1 non structuring, non penetrating		10 (71)		
	B2 stricturing		2 (14)		
	B3 penetrating		2 (14)		
Perianal disease n (%)			3 (21)		
Location	E1 proctitis			0 (0)	1 (5)
UC/IBD-U n (%)	E2 left-sided			2 (12)	5 (26)
	E3 extensive			2 (12)	4 (21)
	E4 pancolitis			13 (76)	9 (47)
Severity n (%)	S1 ever severe			17 (100)	19 (100)
Growth delay n (%)		15 (29)	6 (43)	4 (24)	5 (26)
Surgery n (%)			3 (21)^a^		
Prior biologic medication n (%)	Biologic-naïve	12 (24)	1 (7)	4 (23)	7 (39)
	TNF-α naïve		1 (7)	4 (23)	8 (42)
	Ustekinumab		3 (21)	1 (6)	1 (5)
Medication at vedolizumab induction n (%)	TNF-blocker		13 (93)	13 (77)	11 (58)
	Azathioprine		3 (21)	0 (0)	3 (16)
	Methotrexate		1 (7)	1 (6)	1 (5.3)
	5-ASA		5 (36)	9 (53)	13 (68)
	Corticosteroid		11 (79)	14 (82)	17 (90)
Fecal calprotectin at vedolizumab induction					
median $\mu$g/g		745	826	641	781
(Interquartile range)		(245–1617)	(657–987)	(342–1241)	(781–1216)
< 100 $\mu$g/g		4 (8)	0	1 (6)	3 (16)
> 1000 $\mu$g/g		16 (32)	3 (21)	6 (35)	7 (37)

^a^2 ileocecal resections, 1 sigma resection

The median age of all patients at diagnosis was 12.1 years and 15.3 at first VDZ treatment when disease duration was a median of 2.3 years. Of all patients, 20% (10/50 patients) were diagnosed less than one year before VDZ treatment. The baseline characteristics of the patients are outlined in Table 1.

**Fig. 1:**
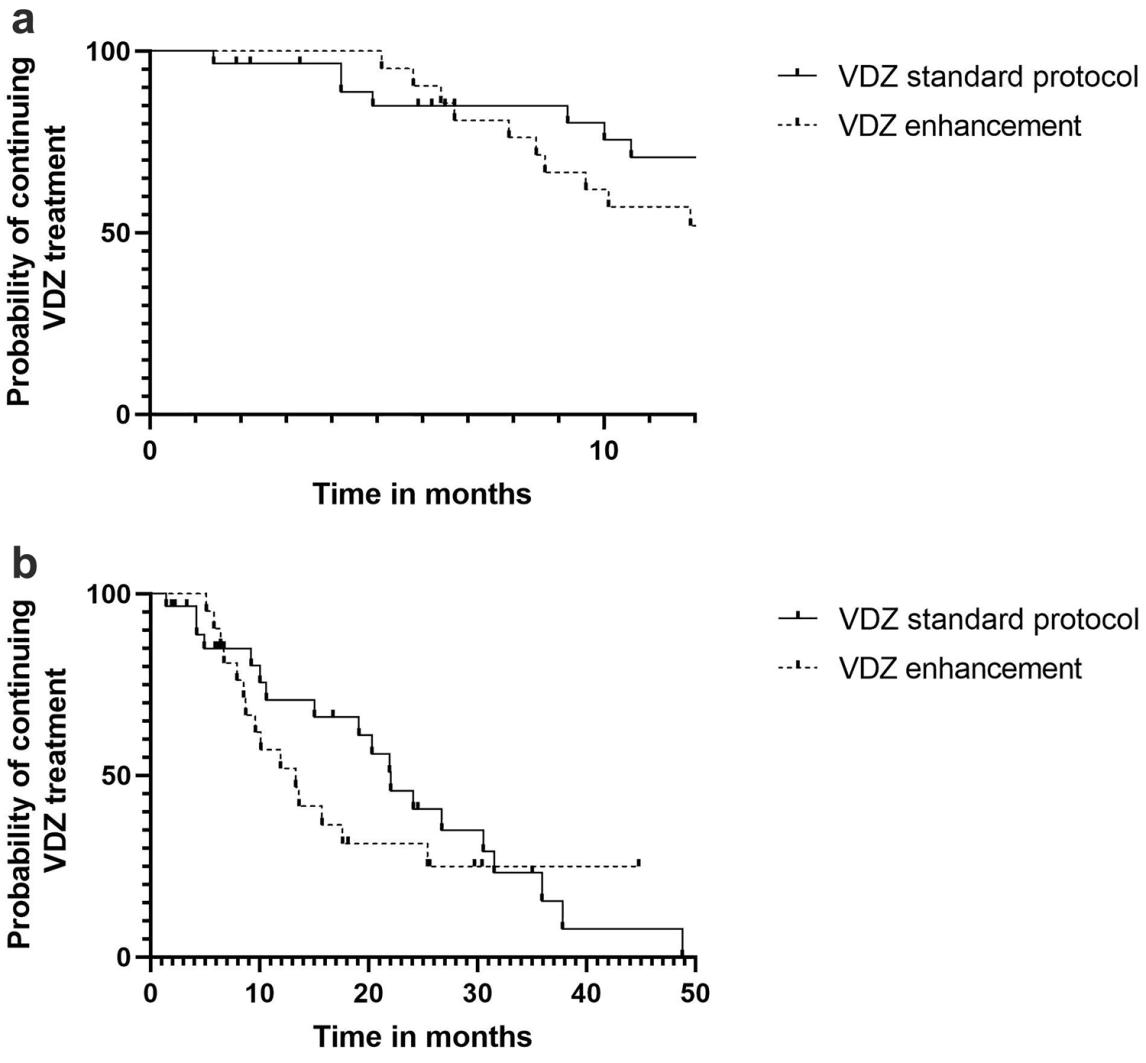
Probability of continuing vedolizumab (VDZ) treatment at first 12 months (p = 0.73;Log-rank test)) (**a**) or during the entire follow-up (p = 0.42; Log-rank test)) (**b**) in pediatric patients with inflammatory bowel disease treated with standard protocol and in patients with treatment enhancement. Of 50 patients, 29 patients followed standard protocol and 21 had treatment enhancement during the follow-up

Of the 50 patients, 13 (26%) discontinued for poor VDZ response (lack of primary response in 9 (18%) patients and disease exacerbation after VDZ induction in 4 (8%)) at a median time of 6.7 months (mean 10 months), and 16 were transferred into adult healthcare while continuing VDZ. There was no discontinuation due to adverse events. Of 50 patients, 80% (40 patients) were on VDZ maintenance therapy at 6 months and 50% (25 patients) at one year (Fig. 1). The specific IBD diagnoses (CD, UC, or IBDU) were not associated with the discontinuation of VDZ for lack of response (p = 0.57).

### VDZ dose and therapy intensification

In 42 patients VDZ was introduced as 300 mg (corresponding to a mean of 5.1 mg/kg) In 8 patients the first VDZ dose was lower from 100 to 250 mg (corresponding to a mean of 5.4 mg/kg) and further doses increased to correspond with weight.

At 3 months, 21% (10 out of 47 children still on VDZ treatment) had therapy intensification and received one additional VDZ dose compared to others. At 6 months 48% (19 of 40 children) had therapy intensification and received 1 to 3 doses more VDZ than those following the standard 8-week-maintenance regimen. Patients who received VDZ as standard protocol were as likely to continue VDZ treatment at 12 months as patients with VDZ treatment intensification (Fig. 1a and b, and Additional Table 1), although at 6 months there seemed to be a minor difference (80% on maintenance in the group undergoing intensification versus 70% in the group with no intensification). No difference in achieved CF was observed between patients with standard protocol and treatment intensification at 12 months (58% and 52%, respectively, p = 0.716). The baseline characteristics of the patients treated according to standard protocol or with enhanced therapy were comparable (Additional Table 2, no statistically significant differences between the groups).

### Disease duration and prior anti-TNF treatment

Patients introduced to VDZ within the first year after PIBD diagnosis (1 CD patient, 7 UC patients, and 2 IBD-U patients) discontinued VDZ for primary lack of response or loss of response numerically more often (5/10 patients; 50%) than patients who received VDZ after the first year of disease (8/40 patients; 20%) (p = 0.067). Anti-TNFα -naïve patients were as likely to discontinue VDZ for lack of response (n = 4/13, 30.7%) than those with previous anti-TNFα treatment (n = 10/37, 27%, p = 0.79).

### VDZ trough levels

For all patients, the mean VDZ trough level was 39.3 μg/mL (IQR 25-47 μg/mL) at week 6, 30.5 μg/mL (IQR 19-41 μg/mL) at 3 months (n = 46), and 25.6 μg/mL (IQR 21-31 μg/mL) at 6 months (n = 46). VDZ trough levels were similar in all time points despite of IBD type (CD, UC, or IBD-U, data not shown). In patients with a weight under 40 kg (N = 5), the VDZ dose/kg was slightly higher (6.2 mg/kg) but trough levels were numerically lower (34 μg/mL, IQR 22-47 μg/mL) than in those over 40 kg (N = 45; VDZ 4.9 mg/kg; 41.3 μg/mL, IQR 21-38 μg/mL, respectively), however, the differences were not statistically significant.

Although children with early treatment with VDZ (during the first year after diagnosis) seemed to have numerically higher trough levels at 6 weeks and 3 months compared to those with longer duration of disease (43.8 μg/mL (IQR 21-58 μg/mL) vs. 38.0 μg/mL (IQR 27-39 μg/mL) at 6 weeks, and 36 μg/mL (IQR 25-47 μg/mL) vs. 29 μg/mL (IQR 17-43 μg/mL) at 3 months, respectively), the differences were not statistically significant (p = 0.26 and p = 0.11, respectively).

VDZ trough levels were not associated with the lack of response. Patients who discontinued for lack of response had VDZ trough levels 36.4 μg/mL (IQR 30-52 μg/mL) at baseline after the first dose, 33.0 μg/mL (IQR 27-40 μg/mL) at 3 months and 26.8 g/mL (IQR 18-40 μg/mL) at 6 months. In children who continued VDZ, the trough levels were 40.2 μg/mL (IQR 25-47 μg/mL) after the first dose, 29.6 μg/mL (IQR 16-45 μg/mL) at 3 months, and 26.6 μg/mL (IQR 22-32 μg/mL) at 6 months (p = 0.54, p = 0.39, p = 0.98, respectively).

### VDZ drug antibodies

VDZ antibodies were measured from all samples but not detected in any sample.

### Corticosteroid-free therapy

**Fig. 2:**
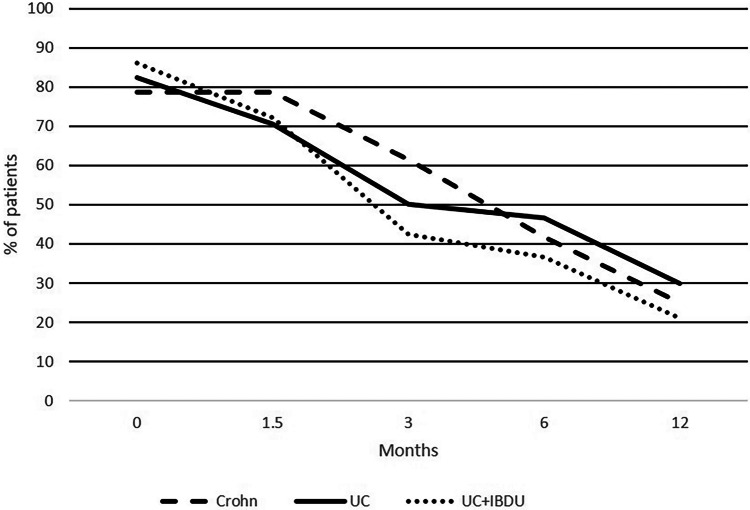
The proportion of patients using corticosteroid therapy and vedolizumab in pediatric patients with Crohn’s disease (N = 11), ulcerative colitis (UC) (N = 14), and unclassified inflammatory bowel disease (IBD-U) (N = 17). Patients without corticosteroids at baseline have been excluded

Before VDZ initiation, 42 (84%) children were using corticosteroids at baseline. Of these, 6 discontinued the VDZ therapy during the first 6 months for lack of response. CF maintenance therapy was achieved in 41% (16/39 patients) at 3 months, in 52.7% (19/36 patients) at 6 months, and in 77% (17/22 patients) at one year. Rates of achieved CFs were similar in all PIBD types (Fig. 2 and Additional Table 3) (p = 0.88).

**Fig. 3:**
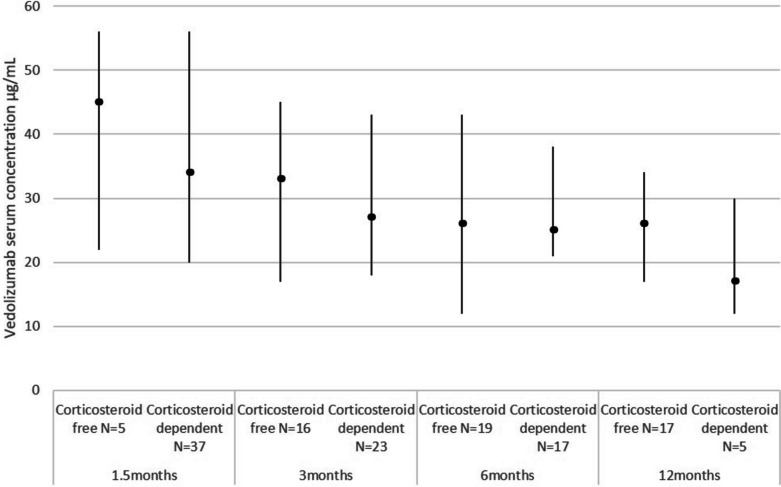
Vedolizumab trough levels in corticosteroid-free and corticosteroid-dependent pediatric patients with inflammatory bowel disease at 1.5 months (6 weeks), 3 months, 6 months, and 12 months. A vertical line defines the interquartile range, the median is marked as a square

VDZ trough levels did not associate with the achieved CF maintenance therapy (at 6 weeks p = 0.09, at 3 months p = 0.12, at 6 months p = 0.93) (Fig. 3). At 3 months, patients achieving CF and those dependent on corticosteroids had comparable trough levels (a median level of 33.1 μg/mL (IQR 29-41 μg/mL) vs 27.5 μg/mL (IQR 16-38 μg/mL), respectively). One of eight patients without corticosteroids at baseline was introduced to corticosteroids before the end of follow-up but was not included in the above-mentioned analyses.

### Fecal calprotectin

**Fig. 4:**
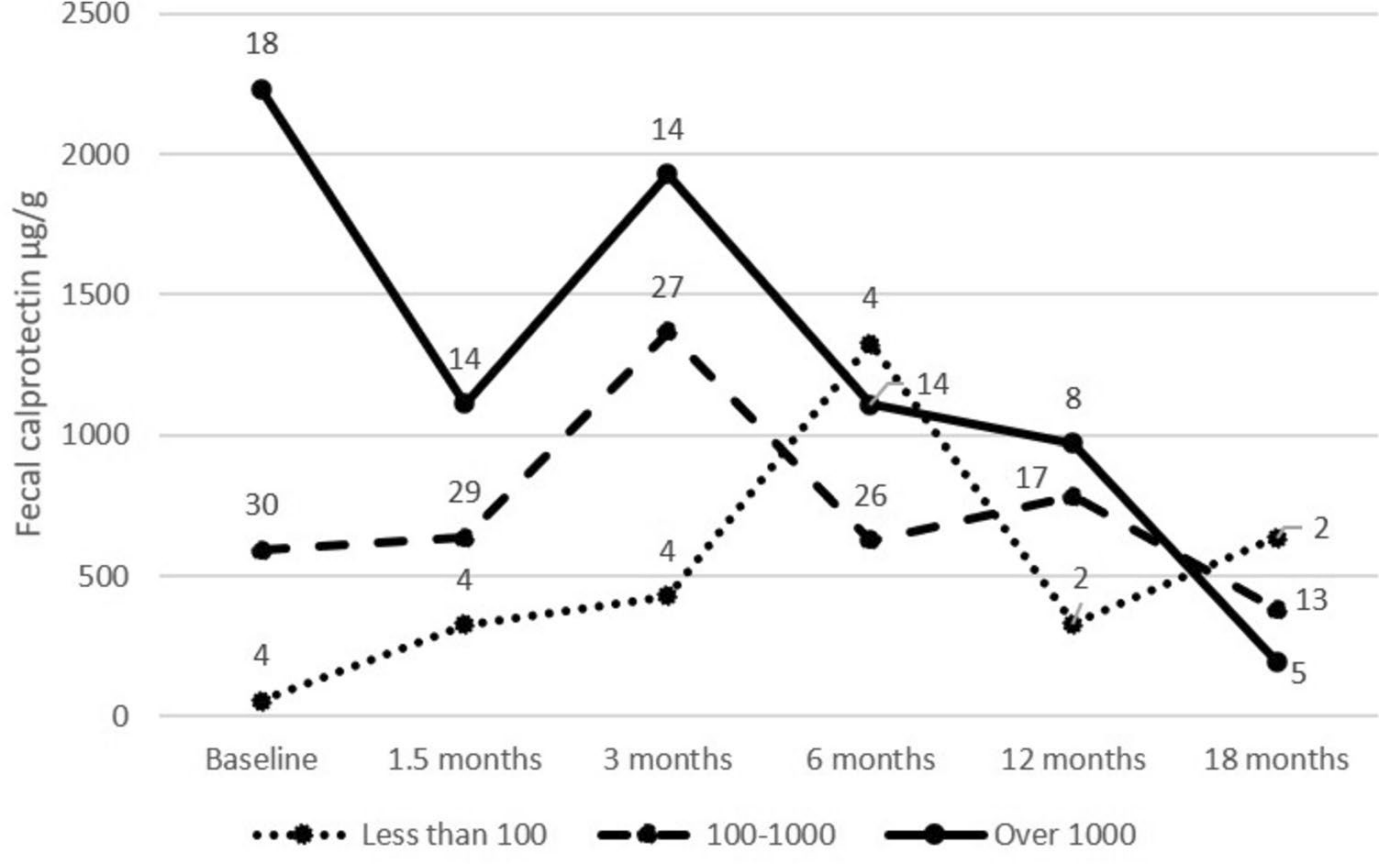
Fecal calprotectin during vedolizumab treatment is categorized according to baseline levels at therapy initiation (< 100 μg/g, 100–1000 μg/g, and > 1000 μg/g) in pediatric patients with inflammatory bowel disease. The number of patients and the mean values of fecal calprotectin marked in each time point

At baseline, the median FC levels were similar in patients with CD compared to patients with UC or IBD-U (Table 1; p = 0.48 and p = 0.47, respectively) showing active disease. At baseline, FC was < 100 μg/g (defining remission) in 4 patients (steroid dependents) (Table 1). The pattern of FC levels during the VDZ therapy is shown in Fig. 4. At 3 months there was no difference in the trough levels between groups in remission with FC < 100 μg/g (mean VDZ 36.8 μg/mL, IQR 32-47 μg/mL, n = 11) or active disease FC > 100 μg/g (mean VDZ 27.4 μg/mL; IQR 13-43 μg/mL, p = 0.0698) or when categorized in the three FC groups: FC < 100, FC 100–1000 μg/g (mean VDZ 28.6 μg/mL, IQR 17–38 μg/mL, n = 22) or FC > 1000 μg/g (mean VDZ 27 μg/mL, IQR 14–41 μg/mL, n = 9) (p = 0.189, Kruskall-Wallis). The pattern of VDZ concentrations throughout the 12-month follow-up period related to the observed FC values is shown in Additional Fig. 1.

FC decreased at 6 weeks in those who continued with VDZ (from a median of 761 μg/g (IQR 244–1251 μg/g) at baseline to a median of 313 μg/g (IQR 34-761 μg/g) at 6 weeks, p = 0.026). In patients discontinuing VDZ at any point, the median FC did not decrease during the first 6 weeks (at baseline 728 μg/g (IQR 605–1216 μg/g) and 742 μg/g (IQR 183–3240 μg/g) at week 6).

FC increased during the follow-up in 16/50 (32%) patients. VDZ trough levels were similar in patients with a decreasing calprotectin value and with patients with constant or increasing FC during the follow-up (p = 0.18 at 6 weeks, p = 0.97 at 3 months, and p = 0.74 at 6 months).

### Adverse events related to VDZ

There were no treatment withdrawals or discontinuances due to adverse events.

## Discussion

The study describes a real-life setting of VDZ therapy and trough levels in 50 pediatric patients with IBD during a median follow-up of 12 months. We found that in patients with PIBD, higher trough levels of VDZ did not associate with better treatment outcomes as reflected in the decline of FC levels or with achieving a corticosteroid-free state during maintenance treatment.

VDZ trough levels were not associated with achieved beneficial outcomes, as patients with decreasing FC values at any point or those with CF maintenance had similar trough levels with patients without achieved response in the gut inflammation (a decrease in FC less than 50%). In the pediatric population, the targeted mean VDZ trough levels in clinical practice are derived from clinical GEMINI trials in adults. In these studies, however, the advantages of higher trough levels were contradictory [9, 10, 15, 17]. In our study, no trough level predicting remission or corticosteroid withdrawal could be determined. The trough levels were not associated with the decline of FC levels. The commonly used dose is weight-based averaging between 5 to 6 mg/kg/dose with a maximum at the adult FDA (Food and Drug Administration) approved dose of 300 mg. In adults, the targeted drug levels > 20 μg/ml at week 6 were associated with improved clinical outcomes [22]. In our pediatric cohort, the average dose was comparable, and drug levels were mostly above > 30 μg/ml. In a previous cohort of 22 patients with PIBD, the authors did not find any association between the trough levels and the likelihood of staying on maintenance therapy. The measured mean levels, however, were lower than in our patients (10.5 μg/ml and 16.3 μg/ml in groups continuing or discontinuing the therapy; [15]). In the recent pediatric multicenter study reporting therapy outcomes until week 14, the VDZ concentrations were a median of 24.7 μg/ml at week six but only 9.1 μg/ml at week 14 with no association with CF [23]. These findings support the reports from adult studies that there is no clear exposure-efficacy relationship for VDZ during the maintenance phase. This could partly relate to near complete occupancy of α4β7 integrin in peripheral blood memory T cells regardless of dose [24].

The decision of treatment enhancement was made at the discretion of pediatric gastroenterologists. In PIBD, the treatment intensification of VDZ has led to variable results and only a part of patients benefitted from dose escalation [12]. In our study, 48% of the patients underwent treatment intensification during the first 6 months resulting in a slightly increased proportion (80% vs. 70%) of patients continuing with VDZ treatment compared to standard protocol. However, this benefit disappeared at 12 months. Similarly, Ledder et al. detected no impact on clinical outcome with 4-week vs. 8-week dosing [12].

Of our study population on maintenance, 41% of patients were CF at 3 months and 53% at 6 months. In a previous pediatric study, corticosteroid- and/or exclusive enteral nutrition-free clinical remission was seen in 34% of UC/IBDU patients and 19% of CD patients at 22 weeks (intention to treat analysis) [12]. In another pediatric study, the proportion of patients achieving CF maintenance was higher at 22 weeks (71% of UC patients and in 33% of CD patients) [13]. We did not detect any difference between UC or CD patients but notably, most of our patients with CD (76%) had ileocolonic disease. In line with this, Singh et al. reported that patients with colonic disease (UC or isolated Crohn´s colitis) were more likely to reach remission than patients with non-colonic PIBD [13].

In our study, most children had received either anti-TNFα or other biologic therapy before the start of VDZ. Still, 24% were naïve for any biologic treatment allowing also to study the relation of early start of VDZ to clinical outcome. In our study, the response to VDZ in patients with prior TNF antagonist failure was comparable to that in TNF-naïve patients. Thus, although some studies have suggested that early treatment with biologic agents could improve the clinical outcomes [25] our results did not favor this hypothesis for VDZ in PIBD.

For VDZ, there are only a few potential predictors of favorable response such as the expression of α4β7 in blood in VDZ responders or high levels of circulating IL-6 in non-responders [26, 27]. However, the value of these markers is debated, and these assessments were not available for our patients.

In the GEMINI long-term study [28], 81% of adult patients with clinical response at 6 weeks, were still in clinical remission after 52 weeks. Disease activity was assessed with a partial Mayo score and FC was not measured. We observed that a decline in FC level at 6 weeks was a predictor of staying on VDZ maintenance for at least 12 months. Previous studies on anti-TNFα have shown accordingly that lower FC levels after induction therapy are associated with improved long-term prognosis of therapy [29–31].

In this study, the reasons for VDZ withdrawal were either lack of response or disease exacerbation after induction therapy. There were no treatment-related serious adverse events such as deaths, allergic reactions, or other severe complications. The safety profile of VDZ is considered favorable with no alarming signs of infections, malignancies, or hepatic events [32].

VDZ antidrug antibodies were not detected in any sample in our population. This is similar to the findings in a smaller pediatric cohort [15]. The VDZ antidrug antibodies from the GEMINI 1 or 2 population were recently analyzed using a drug-tolerant electrochemiluminescence assay [33]. The VDZ antidrug antibodies were detected in 2.4% of continuously treated patients. No relationship was found between immunogenicity and infusion-related reactions. Based on this, we did not expect to find antidrug antibodies as our study only included patients introduced to VDZ for the first time.

The strength of our study is the comprehensive inclusion of all patients with PIBD treated at our hospital during the study period and all patients having trough level measurements based on the routine follow-up. As a limitation of the study like in most pediatric studies, the number of included patients is relatively low and heterogeneous. As this is a real-life setting, treatment protocols varied. In pediatric patients, endoscopy is considered invasive and less used than in adults and we could not report endoscopy outcomes. Importantly, fecal calprotectin values were available providing additional information on disease activity. Also, our study population represented teenagers with a median age over 12 years and weight over 50 kg, with only a few smaller children that could present with different pharmacokinetics of the drug. Reassuringly, severe adverse events were non-existent. Notably, in our study, all outcome results are related to the use of VDZ, and no additional drugs (biologic, azathioprine, or methotrexate) were started for any of the patients during the study period.

Taken together, in a real-life setting in a pediatric population, VDZ was a well-tolerated and safe biological treatment. An early decline in FC was associated with treatment adherence although there were some late responders. Higher VDZ trough levels did not correlate with steroid-free remission. Accordingly, a VDZ trough level association with improved therapeutic outcomes of VDZ treatment could not be determined. Thus, further research is needed to determine whether there is any benefit of VDZ trough level monitoring.

### Supplementary Information

Below is the link to the electronic supplementary material.

Supplementary Additional Figure 1. Fecal calprotectin values (< 100 μg/g, 100–1000 μg/g, and > 1000 μg/g) during follow-up in pediatric patients with inflammatory bowel disease and vedolizumab serum concentration at 6 weeks, 3 months, 6 months, and 12 months of therapy. A vertical line defines the interquartile range, the median is marked as a square. (JPEG 59 KB)

Supplementary file2 (PDF 81 KB)

Supplementary file3 (PDF 27 KB)

Supplementary file4 (PDF 98 KB)

## Data Availability

The article and its supplementary material include the datasets supporting the conclusions.

## List of abbreviations

CD: Crohn disease
CF: Corticosteroid free
FC: Fecal calprotectin
IBD: Inflammatory bowel disease
IBDU: Inflammatory bowel disease unclassified
PIBD: Pediatric inflammatory bowel disease
TNFα: Anti-tumor necrosis factor α
UC: Ulcerative colitis
VDZ: Vedolizumab
