# Supplementary figures and images for: Drug levels of VEDOLIZUMAB in patients with pediatric-onset inflammatory bowel disease in a real-life setting
Source: Eur J Pediatr. 2023 Oct 25;183(1):313–22. doi: 10.1007/s00431-023-05255-y (PMC10858127; doi:10.1007/s00431-023-05255-y)

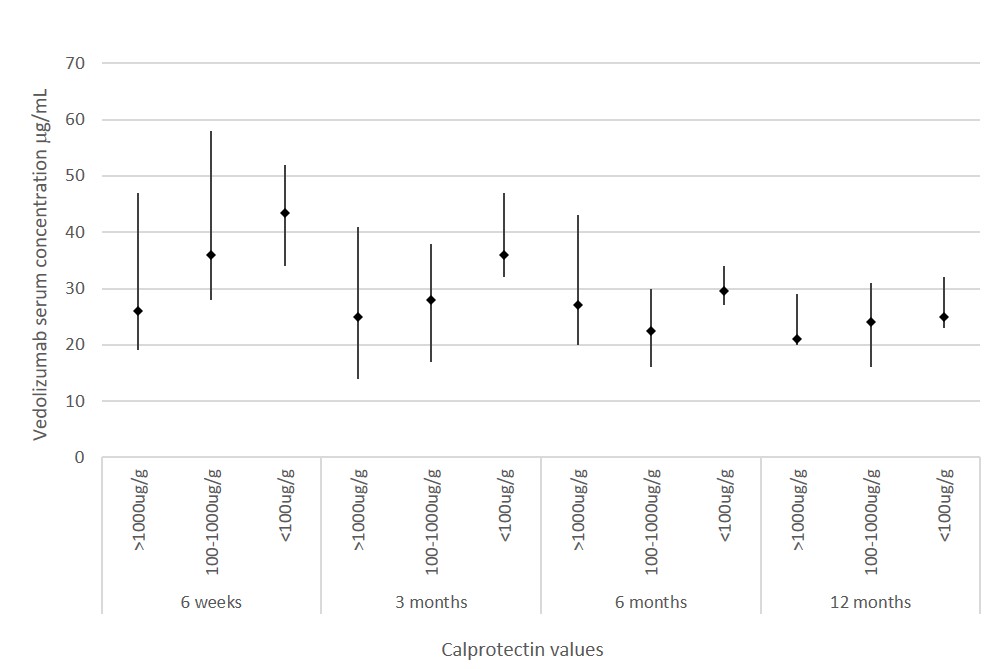

Supplement: Supplementary file 1 — Supplementary Additional Figure 1. Fecal calprotectin values (< 100 μg/g, 100–1000 μg/g, and > 1000 μg/g) during follow-up in pediatric patients with inflammatory bowel disease and vedolizumab serum concentration at 6 weeks, 3 months, 6 months, and 12 months of therapy. A vertical line defines the interquartile range, the median is marked as a square. (JPEG 59 KB) [file 431_2023_5255_MOESM1_ESM.jpeg]
