# Supplementary material for: Drug levels of VEDOLIZUMAB in patients with pediatric-onset inflammatory bowel disease in a real-life setting
Source: Eur J Pediatr. 2023 Oct 25;183(1):313–22. doi: 10.1007/s00431-023-05255-y (PMC10858127; doi:10.1007/s00431-023-05255-y)
Supplement: Supplementary file 2 — Supplementary file2 (PDF 81 KB) [file 431_2023_5255_MOESM2_ESM.pdf]

Number of patients per month															
Months	0	1	2	3	6	9	12	15	18	21	24	30	36	42	48
VDZ standard protocol N=29	29	29	27	26	21	18	15	15	14	12	10	6	2	2	1
VDZ enhancement protocol N=21	21	21	21	21	19	14	11	9	7	6	6	2	2	1	0
