# Supplementary material for: Drug levels of VEDOLIZUMAB in patients with pediatric-onset inflammatory bowel disease in a real-life setting
Source: Eur J Pediatr. 2023 Oct 25;183(1):313–22. doi: 10.1007/s00431-023-05255-y (PMC10858127; doi:10.1007/s00431-023-05255-y)
Supplement: Supplementary file 3 — Supplementary file3 (PDF 27 KB) [file 431_2023_5255_MOESM3_ESM.pdf]

Additional Table 2. Characteristics of the pediatric patients with standard VDZ protocol and enhancement protocol

		Standard protocol (N=29)	Enhancement protocol (N=21)
Age at diagnosis, years, median (range)		11.0 (3.2-16.1)	12.4 (3.8-15.5)
Age at start of VDZ, median (range)		15.1 (7.6-19.1)	14.8 (4.6-15.6)
Disease duration, years, median (range)		4 (0.4-12.6)	2.3 (0.3-10.6)
Disease type	Crohn's disease N (%)<br>Ulcerative colitis N (%)<br>IBD-U N (%)	9 (31)<br>8 (28)<br>12 (41)	5 (24)<br>9 (43)<br>7 (33)
Location CD, n (%)	L1 distal 1/3 ileum ± cecal disease<br>L2 colitis<br>L3 ileocolitis<br>L3+L4a upper disease	0<br>1 (11)<br>5 (56))<br>3 (33)	0<br>2 (40)<br>1 (20)<br>2 (40)
Behaviour CD, n (%)	B1 non structuring, non penetrating<br>B2 stricturing<br>B3 penetrating	7 (78)<br>1 (11)<br>1 (11)	3 (60)<br>1 (20)<br>1 (20)
Perianal disease n (%)		1 (11)	2 (40)
Location UC/IBD-U n (%)	E1 proctitis<br>E2 left-sided<br>E3 extensive<br>E4 pancolitis	1 (5)<br>4 (20)<br>3 (15)<br>12 (60)	0 (0)<br>3 (19)<br>3 (19)<br>10 (62)
Severity n (%)	S1 ever severe	20 (100)	16 (100)
Growth delay n (%)		9 (31)	6 (29)
Surgery n (%)		2 (7)	1 (5)
Prior biologic medication n (%)	Biologic-naïve<br>TNF-α naïve<br>Exposure for ustekinumab	9 (31)<br>9 (31)<br>2 (7)	3 (14)<br>4 (19)<br>3 (14)

Medication at vedolizumab induction n (%)	TNF-blocker<br>Azathioprine<br>Methotrexate<br>5-ASA<br>Corticosteroid	20 (69)<br>3 (10)<br>3 (10)<br>17 (59)<br>24 (83)	21 (100)<br>3 (14)<br>0 (0)<br>10 (48)<br>18 (86)
Fecal calprotectin at vedolizumab induction<br>median μg/g (25-75% interquartile)<br>< 100 μg/g<br>>1000 μg/g		781 (492-1721)<br>3 (10)<br>9 (31)	684 (130-1251)<br>1 (5)<br>7 (33)
