# Supplementary material for: Drug levels of VEDOLIZUMAB in patients with pediatric-onset inflammatory bowel disease in a real-life setting
Source: Eur J Pediatr. 2023 Oct 25;183(1):313–22. doi: 10.1007/s00431-023-05255-y (PMC10858127; doi:10.1007/s00431-023-05255-y)
Supplement: Supplementary file 4 — Supplementary file4 (PDF 98 KB) [file 431_2023_5255_MOESM4_ESM.pdf]

Months	Crohn			UC
	Number of patients with corticosteroid	Number of patients	% of patients on corticosteroid of all patients	Number of patients with corticosteroid
**Beginning of VDZ**	11	14	0,785714286	14
**1.5 months**	11	14	0,785714286	12
**3 months**	8	13	0,615384615	8
**6 months**	5	12	0,416666667	7
**12 months**	2	8	0,25	3

		IBDU		
Number of patients	% of patients on corticosteroid of all patients	Number of patients with corticosteroid	Number of patients	% of patients on corticosteroid of all patients
17	0,823529412	17	19	0,894736842
17	0,705882353	14	19	0,736842105
16	0,5	7	17	0,411764706
15	0,466666667	5	15	0,333333333
10	0,3	2	9	0,222222222
